# Primary Axillary Nerve Injury: A Review of Diagnosis and Management

**DOI:** 10.1186/s13018-026-07148-w

**Published:** 2026-08-07

**Authors:** Joseph DiCecco, Adam Maestas, Christina Brady, Anil K. Dutta

**Affiliations:** UT Health San Antonio, San Antonio, USA

**Keywords:** Axillary nerve injury, Shoulder dysfunction, Glenohumeral dislocation, Brachial plexus, Quadrilateral space syndrome, Electromyography, Nerve transfer, Peripheral nerve reconstruction

## Abstract

Primary axillary nerve injury is a clinically significant yet underrecognized cause of shoulder dysfunction, often leading to substantial morbidity when diagnosis or treatment is delayed. Despite extensive literature on brachial plexus pathology, guidance specific to axillary nerve injury remains limited. Owing to its anatomic course adjacent to the glenohumeral joint, proximal humerus, and quadrilateral space, the axillary nerve is particularly susceptible to traction, compression, and direct trauma. Classic presentation includes deltoid weakness and sensory deficits over the lateral shoulder; however, diagnosis is frequently obscured by concomitant conditions such as rotator cuff tears, fractures, or instability. This review synthesizes current understanding of axillary nerve anatomy, injury mechanisms, diagnostic strategies, and management principles. Common etiologies include glenohumeral dislocation—the most frequent cause—as well as proximal humerus fractures, upper brachial plexus traction injuries, ballistic trauma, and quadrilateral space syndrome. Each entity demonstrates distinct pathophysiology and clinical features, ranging from transient neurapraxia to complete nerve disruption. Accurate diagnosis relies on a combination of clinical evaluation, imaging, and electrodiagnostic studies. Magnetic resonance imaging and MR neurography are increasingly utilized but may yield false positives, while electromyography and nerve conduction studies remain essential for lesion characterization and prognostication. Electrodiagnostic testing is particularly valuable in differentiating neurapraxia from axonotmesis and neurotmesis and guiding operative decision-making. Most axillary nerve injuries demonstrate a high rate of spontaneous recovery with nonoperative management because most cases maintiain nerve continuity. However, absence of clinical or electrodiagnostic improvement by 3–4 months warrants surgical consideration. Operative strategies include neurolysis, nerve grafting, and nerve transfer, with timing tailored to injury severity and mechanism. Early intervention is critical in cases of confirmed transection or high-grade injury to optimize reinnervation before irreversible motor end plate degeneration. Quadrilateral space syndrome represents a distinct compressive neuropathy with both neurogenic and vascular subtypes, typically managed conservatively but occasionally requiring surgical decompression. Early recognition, appropriate diagnostic workup, and individualized management are essential to optimize functional outcomes in patients with primary axillary nerve injury.

## Background

Primary axillary nerve injury is an important cause of shoulder dysfunction and may result in substantial morbidity when diagnosis or treatment is delayed. Even so, while there are many papers describing brachial plexus injury, there is a paucity of literature specifically guiding diagnosis and management of isolated axillary nerve injury. Owing to its intimate relationship with the glenohumeral joint, proximal humerus, and quadrilateral space, the axillary nerve is particularly vulnerable to traction, compression, and direct trauma in a variety of clinical settings. Although injury classically presents with deltoid weakness and sensory loss over the lateral shoulder, the diagnosis is often obscured by concomitant pathology such as rotator cuff tear, fracture, or glenohumeral instability. As a result, axillary nerve lesions may be underrecognized in both acute and chronic presentations.

The spectrum of axillary nerve injury ranges from transient neurapraxia with a high likelihood of spontaneous recovery to more severe lesions requiring surgical reconstruction. Common etiologies include brachial plexus traction injuries, glenohumeral dislocation, proximal humerus fracture, and quadrilateral space syndrome (QSS), each with distinct mechanisms, clinical features, and management considerations. Advances in electrodiagnostic testing, magnetic resonance imaging (MRI), and operative nerve reconstruction have improved the ability to characterize these injuries and tailor treatment to the severity and chronicity of the lesion.

This review summarizes the anatomy of the axillary nerve, the major etiologies and clinical presentations of primary axillary nerve injury, the role of diagnostic imaging and electrodiagnostic studies, and current principles of nonoperative and operative management.

## Relevant Anatomy

Understanding the anatomy of the brachial plexus and the axillary nerve’s course through the shoulder joint contextualizes the causes and treatments of injury. The brachial plexus originates from ventral spinal roots C5, C6, C7, C8, and T1. Axillary nerve fibers originate from ventral spinal roots C5 and C6. The five roots converge into upper, middle, and lower trunks supraclavicularly. The upper plexus is most superficial as it approaches the clavicle and is referred to as Erb’s point [1]. Here, the plexus is most vulnerable to blunt force injury. As the plexus courses posteriorly to the clavicle and superior to the first rib, the anterior and posterior divisions converge into the lateral, posterior, and medial cords.

**Fig. 1:**
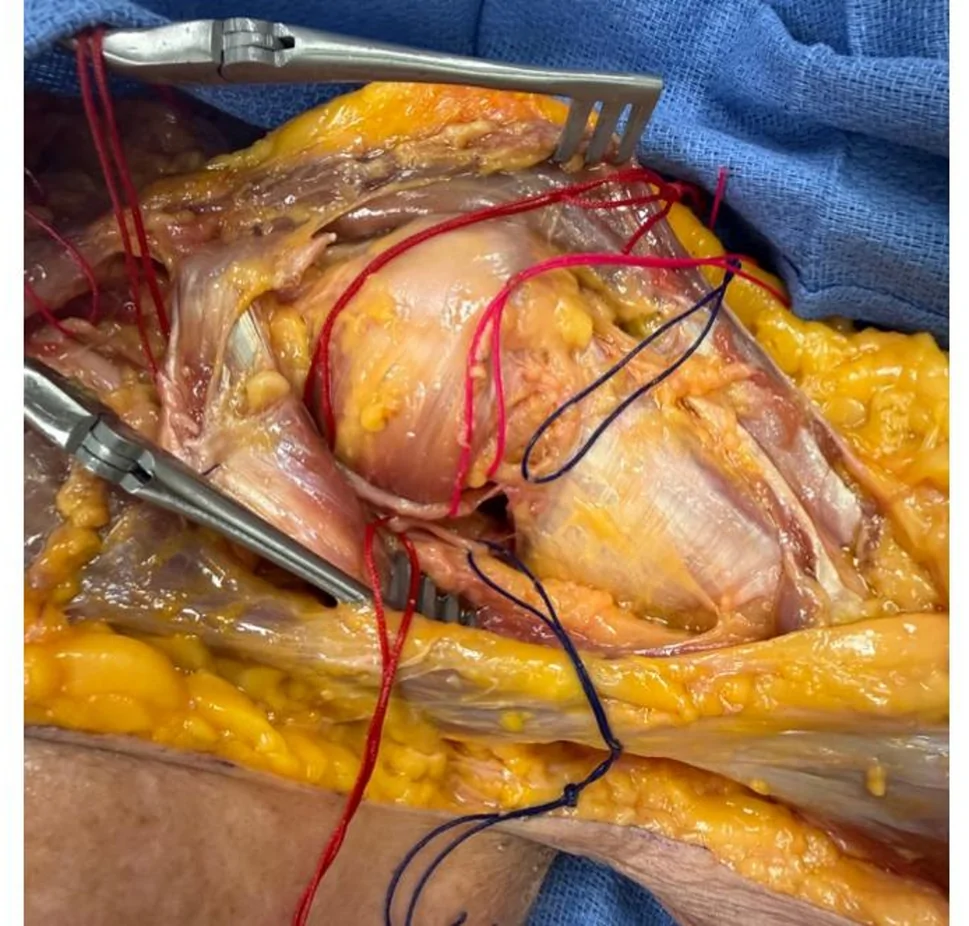
Axillary nerve, anterior humeral view

**Fig. 2:**
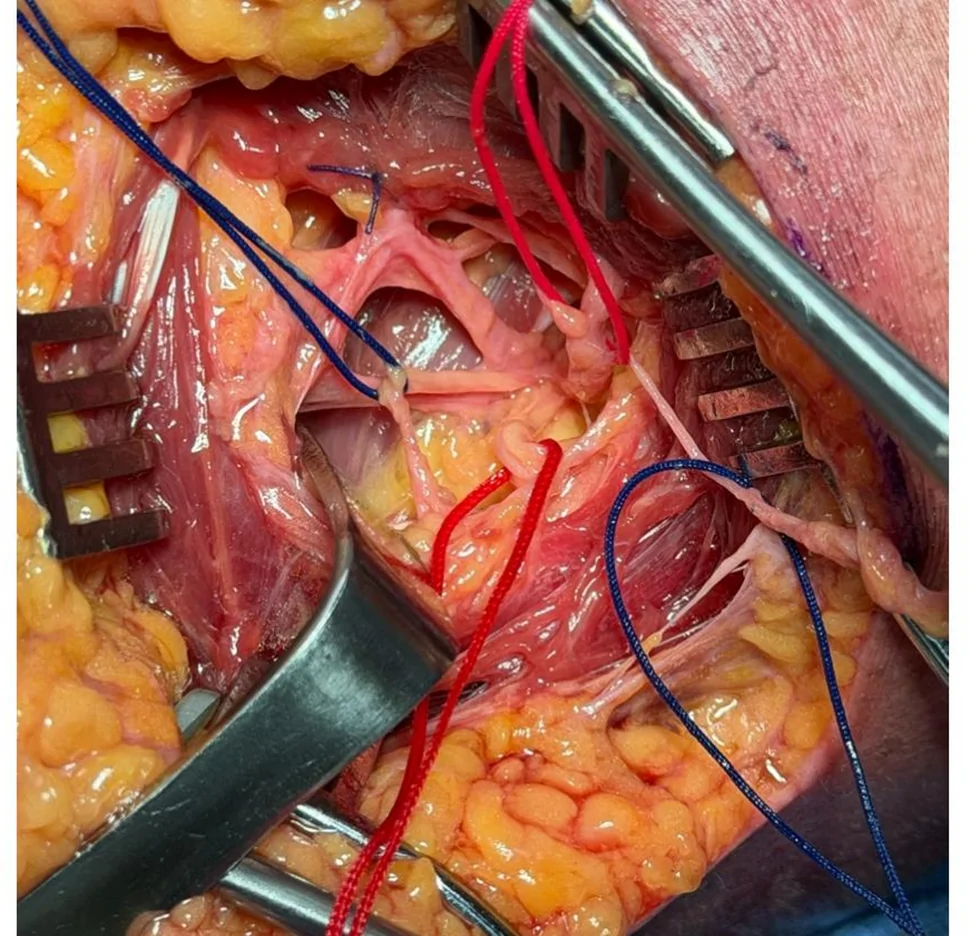
Axillary nerve, split-deltoid view. Branches of the axillary nerve are viewed as they course deep to the deltoid: the cutaneous branch (right blue tag), anterior axillary branch (top red tag), branch to teres minor (bottom red tag), posterior axillary branch (left blue tag)

The axillary nerve proper emerges from the posterior cord and courses inferior to the glenohumeral joint and posteriorly through the quadrilateral space along with the posterior circumflex humeral artery (Figs. 1 and 2). The quadrilateral space is bound superiorly by the teres minor, inferiorly by the teres major, laterally by the surgical neck of the humerus, and medially by the long head of the triceps. The axillary nerve divides into anterior and posterior branches within the quadrilateral space 88% of the time and within the deltoid muscle 12% of the time [2]. After passing through the quadrilateral space, the anterior branch provides motor innervation to the anterior two-thirds of the deltoid, and the posterior branch innervates the posterior third of the deltoid and continues as the superior lateral brachial cutaneous nerve, supplying sensation to the “regimental badge” area overlying the deltoid [1–3]. The posterior branch of the axillary nerve becomes susceptible to direct trauma during its superficial course in the deep subfascial surface of the deltoid muscle [4]. Only one branch of the nerve supplies the teres minor, but many branches supply the deltoid muscle. Its free course is short; this allows little margin for stretching under traumatic stress [5].

## Etiologies, Presentation, and Imaging

Axillary nerve injury classically presents with deficits in abduction (especially from 15° to 90°) and external rotation owing to deficits from the deltoid and teres minor, respectively. Additionally, sensation is lost in the deltoid region in the axillary nerve distribution (“regimental badge” region) [6]. Complete loss of abduction may be more indicative of rotator cuff tear than axillary nerve injury, although these injuries can mask each other in the case of an unhappy triad [5, 7]. These findings are not specific to axillary nerve injury and physical exam alone may not differentiate between rotator cuff tear and nerve injury [7]. The clinician should be aware of other injuries that may present similarly to axillary nerve palsy, such as suprascapular nerve injury, C5 and/or C6 compression, and Parsonage-Turner Syndrome (neuralgic amyotrophy). Therefore, diagnosis may be aided by imaging technology. MRI is increasing in popularity due to new techniques and high accuracy, although it is liable to false-positive interpretations [8].

Electrodiagnostic studies, such as nerve action potential, are particularly useful intra-operatively in discerning complete from incomplete lesions, and viable fascicles from non-viable fascicles [8]. The diagnosis of non-iatrogenic axillary nerve injury can be divided into four etiologies: upper brachial plexopathy, glenohumeral dislocation, QSS, and proximal humerus fracture.

### Glenohumeral Dislocation

Glenohumeral dislocation is the most common cause of axillary nerve injury, with an incidence of 1–2% [3, 9, 10]. The most common dislocation direction is anterior, occurring during forceful abduction and external rotation [3, 9]. The axillary nerve is the most frequently affected nerve in shoulder dislocation, occurring in 6–35% of dislocations [3, 10–13]. Dislocation has a bimodal distribution, split into adolescents or young adults suffering high energy trauma and middle-aged or elderly patients suffering low-energy falls. Neurologic deficit is more likely when concomitant rotator cuff tears or greater tuberosity fractures are present [12]. John Stevens first described that anterior dislocation causes axillary nerve injury by stretching the nerve across an abducted and externally rotated humeral head [5, 7]. As the humeral head is forced into the subcoracoid space, the traction force is responsible for the nerve injury [9]. Extreme abduction and internal rotation can also cause axillary nerve injury by wedging the plexus and opening the scapulohumeral angle, causing longitudinal traction on the nerve [3, 5, 7].

While traction force is the main mechanism of axillary nerve injury due to shoulder dislocation, secondary hematoma formation can also cause delayed nerve injury. An expanding hematoma causes compression and stretching over days to weeks, ultimately leading to scarring and adhesions [9]. Vascular injury can also cause formation of pseudoaneurysm or false aneurysm of the axillary artery. Both hematoma and aneurysm formation are characterized by normal nerve function at the time of injury followed by gradual deterioration of function [7]. Vascular injuries occur in 21% of traumatic brachial plexus injuries, with axillary and subclavian vessels being most common [14]. Associated fractures or dislocations increase the odds of concomitant vascular injury by 40%.

### Ballistic Injury

The mechanism of ballistic axillary nerve injury is unique from other mechanisms due to the degree of energy involved. While both low-velocity weapons (pistols, shotguns, explosive devices) and high-velocity weapons can directly transect the axillary nerve, higher velocity weapons are more likely to damage the nerve by cavitation force because they impart a larger zone of injury [15]. Cavitation injury rarely leads to complete loss of continuity, and minority of ballistic nerve injuries result in complete transections. A focused history and physical exam should attempt to correlate velocity of the projectile and location of entry and exit wounds with potential extent of nerve injury. A thorough assessment of limb perfusion is also warranted. The decision for early exploration of a ballistics injury can often be difficult based on exam alone. High velocity injuries warrant early exploration, however, lower velocity injuries are not as clear-cut. High quality magnetic resonance (MR) neurography may help identify continuity of nerves [15]. However, retained bullet fragments may not allow for MRI use. In the experience of the authors, there have been cases of low velocity injuries causing nerve transection, so close follow up is recommended if the patient is managed nonoperatively.

### Stinger Injuries Involving the Axillary Nerve

While injuries of the upper brachial plexus do not involve axillary nerve proper, the axillary nerve’s fibers run through C5 and C6. Upper plexus injuries occur proximal to the clavicle. Commonly referred to as “stingers,” “burners,” or “burner syndrome,” they are most often caused by contact sports such as football and wrestling [1]. Up to 65% of collegiate football players have reported experiencing stinger injuries [16]. Stingers are caused by either direct compression of the plexus ipsilateral to the impact or traction contralateral to the contact due to whiplash effect. Stingers are typically experienced as acute and temporary numbness, tingling, and pain radiating down the affected extremity immediately after a blow to the upper shoulder or lateral neck. These symptoms typically last less than a minute, but reinjury is likely to occur and can eventually cause long-term nerve damage. While any roots of the brachial plexus may be injured by a stinger, axillary nerve fibers are especially susceptible as they have the most superior origins of the brachial plexus (C5, C6) and, as such, have a longer exposed course before diving deep under the clavicle.

### Quadrilateral Space Syndrome

Among axillary nerve pathologies, QSS represents a distinct and underrecognized entity characterized by compression of the axillary nerve and/or the posterior circumflex humeral artery (PCHA) as they traverse the quadrilateral space. QSS was first discovered in 1983 by Cahill and Palmer as the cause of misdiagnosed thoracic outlet syndrome [17]. QSS affects men more than women and affects active patients, particularly overhead athletes, most often. It most often occurs in the dominant shoulder [2].

QSS can be caused by any condition that decreases the cross section of the quadrilateral space and compresses its contents. The most common cause of this is the formation of fibrous bands, which originate from the fascial layer overlying the long head of the triceps and attach to teres major adjacent to axillary nerve in the quadrilateral space. These bands cause a decreased cross-section of the quadrilateral space when they tighten during shoulder abduction, external rotation or internal rotation [2]. Interestingly, one cadaveric study found that fourteen out of sixteen cadavers without prior QSS symptoms had fibrous bands [18]. QSS can also be caused by space occupying lesions such as a paralabral cysts, fracture fragments, benign tumors, axillary schwannomas, venous dilation, or muscle compression in competitive overhead athletes [2]. Overhead athletes are susceptible to QSS because they repeatedly place the shoulder into positions that narrow the quadrilateral space and because the musculotendinous borders of the quadrilateral space (teres minor, teres major, and long head of the triceps) are commonly hypertrophied and/or fibrosed in overhead sports.

While there is no consensus on if the cause of QSS is primarily vascular or neurogenic, a review by Brown et al. broadened the definition of QSS and subdivided it into vascular QSS (vQSS) and neurogenic QSS (nQSS) [19]. They describe vQSS and nQSS as separate, but overlapping entities with different etiologies and presentations. nQSS is most similar to the traditionally held view of QSS: *static* or *fixed* compression of the axillary nerve in the quadrilateral space is responsible for the presenting symptoms. In contrast to nQSS, Brown et al. discusses that vQSS is due to repetitive *dynamic* mechanical trauma caused by turbulent flow and intimal injury. This ultimately leads to thrombotic and embolic consequences in the PCHA of the overhead athlete. These forces affect the PCHA more than the axillary nerve because the axillary nerve is resilient to these dynamic forces due to its elastic quality; it can stretch its length by about 8% [20].

Correct diagnosis of QSS can be challenging, but it has become easier as new diagnostic modalities have emerged. When it was first described, patients would often present with unexplainable pain and paresthesia refractory to non-steroidal anti-inflammatory drugs (NSAIDs) and physical therapy and would subsequently undergo unfruitful tests and treatment [17]. A 2018 study even reports a patient who underwent labral repair, subacromial decompression, exploratory surgery, first rib resection, anterior and middle scalenectomy, neurolysis, pectoralis minor release, acupuncture, physical therapy, and steroid injections before being diagnosed with QSS [21]. nQSS tends to present with tenderness, radicular pain, paresthesias, deltoid fasciculations, and atrophy of the deltoid and teres minor [19, 22]. Teres minor deficits are specific to nQSS. vQSS can present with classic arterial insufficiency symptoms: ischemia of muscles supplied by the PCHA, cyanosis, pulselessness, and pain [22]. Throwing athletes may report pain in the cocking phase of the throw [2]. On physical exam, point tenderness over the quadrilateral space is highly sensitive for QSS [17]. Subclavian arteriograms, as originally used by Cahill and Palmer, are no longer the standard due to poor specificity [2]. Other imaging modalities are used, but each has its drawback. Dynamic arteriography exposes the patient to a high dose of radiation and is expensive. EMG has a low sensitivity due to several converging factors: the compression is often dynamic and positional, the injury is frequently demyelinating or pain-mediated rather than axon-loss, and the key target muscle (teres minor) is technically difficult to accurately needle. MRI and computed tomography (CT) do not provide optimal visualization of the structures. Magnetic resonance angiography (MRA) has low sensitivity and is positive in 80% of asymptomatic individuals. Ultrasound does not detect fibrous bands and has low interobserver reliability [2]. As such, there is no gold standard. Diagnosis must clinically correlate image modality of choice with history and physical exam. McAdams and Dillingham et al. present an algorithm for diagnosis based first on exclusion of other causes [23]. After a history and physical exam with characteristic signs and symptoms, they refer patients to six months of physical therapy to solve possible causes of the presentation. If symptoms continue, they conduct a shoulder MRI. If this does not reveal a source of pain or if there is evidence of QSS such as teres minor atrophy, deltoid atrophy, a quadrilateral space lidocaine block test is performed. A positive lidocaine block test is indicated by pain relief and is diagnostic of QSS. They recommend further confirming QSS with EMG and CT angiography.

### Proximal Humerus Fracture

Proximal humerus fractures are the third most common fracture in adults over 60 years of age [24]. Axillary nerve injury, as diagnosed by EMG, occurs in 6.2–67% of proximal humerus fractures [25]. One study of proximal humerus fractures found axillary nerve neuropathy without denervation in 23% of patients (*n* = 7) and axillary nerve denervation in 19% of the patients (*n* = 6) [25]. Of these six injuries, five had a complex fracture of the proximal humerus (AO11B or AO11C).

The shoulder unhappy triad (or “terrible triad”) was first described by Gonzales and Lopez in 1991, and consists of a brachial plexus injury, rotator cuff tear, and glenohumeral dislocation [7]. The unhappy triad is more common in patients over 50 years of age. The most common brachial plexus injury in this triad is isolated axillary nerve injury [5, 26].

## Definitive Management, Outcomes, and Complications

### Non-Operative and Pre-Operative Management

This review will focus on the management of injuries directly related to the axillary nerve only; repair of concomitant injuries, such as glenoid labral tears and proximal humerus fractures, is beyond the scope of the article. Acute management of axillary nerve injury consists of reducing the associated dislocation or fracture in a timely manner. Some studies have found that increased time to reduction led to increased likelihood of multiple nerve outcomes [5, 9, 26–28]. However, others have not found a significant difference in incidence of nerve injury [3, 11, 13]. Most studies report a greater than 75% chance of spontaneous recovery of axillary nerve injury, especially with infraclavicular lesions [7]. Factors that reduce chances of spontaneous recovery are traction injuries, associated fractures, and other associated nerve injuries [29, 30]. Sensory recovery is an early prognostic indicator and usually precedes return of motor function [7].

Electrodiagnostic studies are an important component of prognostication and treatment selection. Baseline EMG can help differentiate neurapraxia from axonotmesis and neurotmesis [31]. Electrodiagnostic studies should be obtained 3–6 weeks after injury to allow Wallerian degeneration to occur [15, 32]. If there is no improvement in EMG by 4 months, operative exploration is indicated. At this timepoint, intraoperative nerve action potentials can determine with a high level of confidence which nerves are regenerating [15]. Good prognosis for nerve recovery is indicated by the emergence of nascent units, the emergence of early potentials, and a decrease in the quantity of fibrillation potentials.

### Operative Management of Traumatic Lesions

Timing of peripheral nerve reconstruction is of utmost importance, but opinions differ for optimal operative timelines. Operating too early makes it difficult to discern the viability of nervous structures and forgoes the opportunity for spontaneous recovery, which some argue has better results [33]. However, delayed operation may lead to irreversible atrophy and miss the opportunity to re-innervate effector muscles [8].

Ideal timing of surgical intervention remains highly controversial, with difficulties in determining extent of injury adding further complexity. However, the mechanism of injury provides important context for operative timeline. Early operative reconstruction should be strongly considered in injuries with confirmed nerve discontinuity caused by sharp transection or ballistic injury [7, 15]. In these injuries, improvement is highly unlikely, and brachial plexus exploration and repair should be performed three to four weeks after injury [8]. Some authors even argue for repair within 72 h in cases where nerve transection is certain, because nerve retraction and operative trimming can be minimized, potentially avoiding the need for graft repair [33]. Additionally, exploration within 72 h allows use of an intraoperative nerve stimulator to evaluate regenerating nerves. Early operative intervention can also be considered in open cases of preserved nerve continuity without signs of regeneration clinically or on EMG. Pena Larrea et al. found no differences in EMG after surgery compared to the EMG prior to surgery. The first signs of reinnervation can be seen two months after neurolysis and three months after grafting. Regeneration is typically complete after 6–18 months [25].

Operative intervention within three months of the initial trauma allows early reinnervation before end plate degeneration, prevents irreversible changes in effector muscles, alleviates pain, prevents neuroma formation, and prevents compressive perineural scarring [7, 8]. Optimal time frame for surgical reinnervation in closed brachial plexus traction injuries is 6–9 months [8]. After 12–18 months, reinnervation is less likely to be successful.

Preoperative and intraoperative characterization of both the type and severity of nerve injury is paramount. Inappropriate excision of a nerve element with regenerative potential or neurolysis in lesions incapable of recovery can result in sub-optimal outcomes. In injuries where continuity is preserved and regenerative nerve action potentials are present, external neurolysis is alone sufficient in most cases. In these situations, functional deficits can usually be attributed to extrinsic fibrotic scarring which compresses the brachial plexus. Meticulous microsurgical decompression may improve nerve conduction and limb function when performed early after trauma [7].

Internal neurolysis may be indicated when severe neuropathic pain is present or when intraoperative findings reveal a thickened epineurium producing compression of nerve fascicles and compromise of the vasa nervorum. In contrast, nerve grafting is warranted when nerve continuity is disrupted or when regenerative potentials are absent despite an intact nerve sheath [7]. The sural nerve is the most commonly used donor nerve, though medial antebrachial cutaneous nerve or sensory branch of the ulnar nerve are also used on occasion [34]. Some authors caution against routine resection and grafting during initial exploration, noting that functional improvement in certain injury patterns may depend on delayed axonal regeneration following restoration of nerve perfusion through neurolysis [33].

Neurotization, or nerve transfer, sacrifices function of a less important nerve to restore function to a more important nerve. This method of surgical repair avoids using nerve grafts which has been associated with better outcomes [35, 36]. The radial nerve branch to the long head of the triceps is commonly used for transfer to the axillary nerve, though the branch to any head of the triceps can be used [35]. Dual transfers to both the suprascapular and axillary nerves improve functional abduction [37]. A meta-analysis by Merrell et al. consisting of 123 nerve transfers within one year of injury demonstrated that 73% of patients who underwent nerve transfer for restoration of shoulder abduction achieved 3/5 strength or better, and 26% achieved 4/5 strength or better [37]. Neurotization of spinal accessory nerve to axillary nerve is also a viable option, an interpositional nerve graft may be necessary to reach.

In cases of unresolved deltoid insufficiency due to long-term axillary nerve pathology, pectoralis transfer can also be considered as a salvage option. Upper pectoralis major flaps have been shown to adequately span the length of the native deltoid and improve active range of motion in flexion and abduction [38, 39]. This transfer can even be combined with reverse total shoulder arthroplasty to further address deltoid deficiency [40].

Surgical exploration of the brachial plexus is frequently complicated by distorted anatomy secondary to dense fibrotic scarring, and the presence of concomitant injuries or prior procedures in the axillary region further increases technical complexity. Postoperatively, long-term surveillance is recommended for a minimum of two years and ideally up to five years. When surgical intervention is delayed beyond approximately 18 months from the time of injury, reconstructive alternatives such as tendon transfer, muscle transposition, or arthrodesis should be considered to restore essential limb function [7].

### Management of Quadrilateral Space Syndrome

Conservative measures, such as physical activity modification, physical therapy, transverse friction massage techniques, posterior rotator cuff stretches, and NSAIDs should first be recommended after diagnosing QSS [2, 21]. Perineural steroid injections can also bring pain relief and aid in diagnosis. Most cases resolve with conservative treatment [2]. If after six months conservative measures fail, surgical decompression may be considered [21].

The operative technique begins with a 4 cm incision over the posterior shoulder with the patient in lateral decubitus position [2]. The posterior border of the deltoid is then identified and reflected superolaterally, exposing the quadrilateral space. The axillary nerve and PCHA can then be freed from fibrous adhesions. It is important to observe or palpate a freely gliding nerve and artery after decompression [2, 21]. Patients can usually return to full activity several weeks after surgery [19, 21].

The treatment approach to vascular QSS is different, as its aim is to resolve vascular occlusion. For an acute thrombus, administering thrombolytics may resolve the issue. For a chronic thrombus, arterial ligation or thrombectomy may be performed. Resection or endovascular coiling can treat aneurysm formation. Anticoagulants may also aid treatment [19].

## Conclusions

Primary axillary nerve injury is a heterogenous clinical entity requiring management tailored to injury mechanism, severity, and chronicity. Because presentation is frequently confounded by associated shoulder pathology, a high index of suspicion and systematic evaluation are essential. Electrodiagnostic studies remain the cornerstone of lesion characterization and prognostication, while advanced imaging serves as a valuable adjunct in selected patients. Although most injuries with preserved nerve continuity recover without surgery, failure to demonstrate meaningful clinical or electrodiagnostic recovery should prompt timely referral for peripheral nerve evaluation. Careful patient selection and appropriate timing of neurolysis, nerve grafting, or nerve transfer maximize the potential for functional recovery before irreversible denervation occurs. Continued advances in imaging, electrodiagnostic techniques, and reconstructive strategies will further refine diagnosis and optimize outcomes for patients with primary axillary nerve injury.

## Data Availability

No datasets were generated or analysed during the current study.

## Abbreviations

AO: Arbeitsgemeinschaft für Osteosynthesefragen (AO Fracture Classification)
CT: Computed tomography
EMG: Electromyography
MRA: Magnetic resonance angiography
MRI: Magnetic resonance imaging
MR: Magnetic resonance
NSAIDs: Nonsteroidal anti-inflammatory drugs
PCHA: Posterior circumflex humeral artery
QSS: Quadrilateral space syndrome
nQSS: Neurogenic quadrilateral space syndrome
vQSS: Vascular quadrilateral space syndrome
